# Interaction between bacterial diversity and biogenic amines production in a salted mackerel stored at soft frozen (−7℃–0℃) storage

**DOI:** 10.1002/fsn3.2647

**Published:** 2021-12-24

**Authors:** Zhihua Tao, Weiqi Liu, Qinxia Hu, Xue Wu, Shuying Xie, Hongmei Zhang, Minghui Fu, Jing Yang, Yan Jiang

**Affiliations:** ^1^ Department of Food Science and Engineering Guangdong University of Technology Guangzhou China

**Keywords:** biogenic amines, high‐throughput sequencing technologies (HTS), microorganisms diversity, salted mackerel, structure analysis

## Abstract

The bacterial diversity of salted mackerel “one‐night courtyard” at soft frozen area (−7℃–0℃) storage was studied. The fish samples at 0, 14, 21, 28, and 35 days were analysis for bacterial structure using high‐throughput sequencing technologies (HTS) and biogenic amines using high‐performance liquid chromatography (HPLC). The analysis results of HTS showed that the dominant bacteria species was varied gradually following with storage time. On the 0th, 21st, and 28th days of storage, dominant *Vibrionaceae* was accounting for 71.70%, 59.16%, and 70.68% of the total sequences analyzed, respectively. On the 14th and 35th days, *Shewanellaceae* was the dominant bacterial, accounting for 87.53% and 70.95% of the total sequences analyzed, respectively. In addition, 21st and 28th days, an abundance of Operational Taxonomic Units (OTUs) was top. The dominant bacterial of *Proteobacteria*, *Firmicutes*, was producer of biogenic amines. Furthermore, the analysis results of HPLC shown the total biogenic amines of maximum amount 363.01 mg/kg in the sample of HY.14 lower than 1000 mg/kg of the FDA regulation. The range ability of cadaverine was obvious following with the storage time. Cadaverine was 87.36 mg/kg on the 0th day, and it was maximum amount of 276.89 mg/kg on the 14th days. Putrescine was 20 mg/kg on the 0th day and maximum amount of 55.04 mg/kg on the 28thdays of storage. The tyramine was smallest amount of production, and the largest amount was 38.99 mg/kg on 28th, and the smallest amount was 11.97 mg/kg on 35th. Nevertheless, the maximum amount of histamine was 55.04 mg/kg on the 0th day and about 23.14 mg/kg of histamine was little change from 14th to 35th days of storage. Dominant bacteria affect the change of biogenic amines. The study can help understand the interaction between microbial flora and biogenic amines in the salted mackerel of one‐night courtyard.

## Graphical abstract

The variation trend of cadaverine, putrescine, and tyramine was consistent, first increase and then decrease, and among them, the content variation of cadaverine was obvious. Nevertheless, the amounts of histamine decreased gradually following with storage time. The study can help understand the interaction between microbial flora and biogenic amines in the salted mackerel fish of one‐night courtyard.

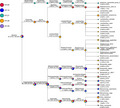

## 1 INTRODUCTION

The salted fish is a traditional processed aquatic product consumed in China. It is popular because of its easy preservation, special flavor, and rich nutrition (Dziezak, 1986). Especially, the salted fish of one‐night courtyard (salted fish pickled overnight) of coastal area in Guangdong of China has a strong fresh flavor. The salted fish of one‐night courtyard belongs to low salt and coarse dry curing products, can eat after 12 hr of pickling time, and maintain the freshness of the fish and keep the fish nutrition, combined with domestic logistics transport developed, the salted fish of one‐night courtyard sells well all over the country, so the eat‐by freshness date is extended to more than one month. Famous fish pickles include salted mackerel, redfish and so on (Wu et al., 2016). The flavor of salted fish is the resultant of fish characteristics and the activity of the microbial communities involved in the pickling process, the salt concentration, and the pickling conditions. The variety of microorganisms involved in the pickling process affect the quality and the flavor of salted fish. The microorganisms such as *Lactic acid bacteria*, *Micrococcus*, and *Staphylococci*, which have enzyme such as proteases, lipases, and amino acid decarboxylases, can promote the decomposition of proteins into amino acids and amino acids convert into biogenic amines or break up fats into short chains of volatile fatty acids and esters (Liu et al., 2016). The metabolic products of microorganisms not only impart a unique flavor to pickled fish products but can also form harmful products (Bianchi et al., 2011). In addition, some *Lactic acid bacteria*, *Micrococcus,* can produce acids and antibiotics, which can inhibit the growth of spoilage bacteria and pathogens such as *Staphylococcus aureus*, *Salmonella*, *Vibrio cholerae*, *Vibrio vulnificus*, *Vibrio parahaemolyticus*, and *Vibrio alginolyticus* in salted fish products (Papageoriou et al., 2018).

Biogenic amines, which are produced by microbial amino acid, decarboxylase to amino acids under favorable conditions, and the salted fish is rich in amino acids. Such as *Escherichia coli* can produce cadaverine from lysine and putrescine from ornithine in the fish products (Pessione et al., 2016). *Lactobacillus curvatus* can also produce histamine from histidine (Singh et al., 2012). The appropriate amount of biogenic amines contributes to normal physiological function within the body, but excessive biogenic amine consumption may lead to death in severe cases due to adverse effects on human health that include hypertension, hypotension, rash, headache, flushing, oral numbness, and digestive problems symptoms of food poisoning (Lee et al., 2012; Wilson et al., 2012).

The formation of useful and harmful substances is closely related to the bacterial structure. However, the structure and composition of microorganisms in crude processing pickled fish of one‐night courtyard of South China are not comprehensive at present and in‐depth understanding. The ecological composition of bacterial communities is one of the most important factors among those responsible for the properties of one‐night pickling salted fishes.

High‐throughput sequencing technology (HTS) is being widely applied to obtain a more comprehensive analysis of microbial diversity (Aldrete‐Tapia et al., 2014; Ercolini et al., 2012). This technique leads to accurate identification of microbial taxa, including those present in low abundance. HTS technology can be carried out by the amplification of 16S rRNA regions coupled with the generation of multimillion reads, and it has enough potential for obtaining a complete coverage of microbial communities, for in‐depth study of the complex interactions between the species present and amount of biogenic amines in a given microbial ecosystem.

The study aimed to analyze the bacteria species involved at different pickling and storing process of salted mackerel until 35 days, and understanding the effect of biological community structure of salted fish on biogenic amines. Biological community structure of salted fish samples was analyzed with HTS analysis conducted by Illumina Miseq of the bacterial 16S rRNA hypervariable regions V3 and V4. The information obtained through HTS approach of microbiology can provide the useful data in understanding the interaction between bacterial flora and biogenic amines, and improving the quality of salted fish.

## 2 MATERIALS AND METHODS

### 2.1 Salting method for fishes based on traditional of one‐night courtyard of South China

The 15 fresh mackerels (about 25 cm, 225 g) were purchased in the same booth from the market of Zhongcun, Panyu, Guangzhou, Guangdong province, China, and send the samples with ice bag to lab in Guangdong University of Technology right now, and put the fresh fishes into refrigerator until use. Then, the fresh mackerels were salted, salting method for fishes based on traditional of one‐night pickling salted fish of South China. All mackerels were washed clean in the laboratory, and some salts (10% of the wet weight of fish) were spread on them. The fishes were kept in a capped, sterilized bottle at room temperature. After 12 hr, the pickled fishes were kept in the soft frozen area (−7℃–0℃) of the refrigerator. Then, samples were collected from day 14 and taken every 7 days, and the microorganisms of the day 0 were used as the base control, so microbial community analyses were carried out after 0 (HY.00), 14 (HY.14), 21 (HY.21), 28 (HY.28), and 35 (HY.35) days of storage.

### 2.2 Microbiological analyses

For the accuracy of the experimental data, 10 g fish meat samples were taken from three parts of the salted mackerel sample, respectively, and crushed and blended together of 30 g sample aseptically, then taking the mixed sample of 10 g, and diluted in 90 ml of sterile peptone water (8.5 g/L NaCl and 1.0 g/L peptone solution at pH 7.0), and crushed to make uniform solutions. Then, 1.0 ml homogeneous liquids were diluted according to the ten‐fold dilution method. Aliquots (0.1 ml) of diluted liquid were spread on the surface of plate count agar (Oxoid, CM0325B) supplemented with 10 g/L NaCl and incubated at 32℃ for 48 hr (Osimani et al., 2011). Each sample was duplicate analyzed. For bacterial counting, lactic acid bacteria (LAB) were grown on pH 5.7 Man‐Rogosa‐Sharpe (MRS) agar (Haibo, Qingdao, China) at 30℃ for 72 hr under anaerobic conditions (ISO 15,214) (Aquilanti et al., 2012); *Staphylococcus spp*. counts were grown on Mannitol‐Salt‐Agar (MSA) medium (Haibo, Qingdao, China) at 37℃ for 48 hr; total *Enterobacteria* (Gram‐negative and cytochrome oxidase negative) were grown on Violet Red Bile Glucose (VRBG) agar (Haibo, Qingdao, China) at 37℃ for 24 hr. Three suitable dilution (10^–6^, 10^–7^, 10^–8^) coated plates were selected. Each dilution was repeated 3 times. The results of the microbial counts were expressed as means of log colony‐forming units (CFU) ± *SD* per gram of sample.

### 2.3 Total bacterial genomic DNA extraction

Total bacterial genomic DNA was extracted directly from the fish sample. Each sample of 250 mg was weighed according to the PowerSoil^®^DNA Isolation kit (Mobio). Extraction of DNA was then carried out based on instructions in TaKaRa MiniBEST Agarose Gel DNA Extraction Kit Ver.4.0 (Takara Biotechnology Co., Ltd). DNA was eluted with TE buffer, and aliquots of 2.0 µl of extracts were also examined by electrophoresis on a 1% agarose gel. Finally, the eluted DNA was stored at −20℃.

### 2.4 PCR amplification and sequencing

The DNA extracted as described above was used to study the bacterial diversity by high‐throughput sequencing of 16S rDNA genes. In detail, PCR amplifications of the bacterial V3‐V4 16S rRNA regions were carried out using the TaKaRa LA Taq^®^ (Takara Biotechnology Co., Ltd) and the primer pairs 341F (5'‐CCTAYGGGRBGCASCAG‐3') and 806R (5'‐GGACTACNNGGGTATCTAAT‐3'). Each PCR mixture was performed using 2.5 µl 10×Ex Taq Buffer, 1 µl dNTP (2.5 mM), 1 µl each of primer, 0.5 µl rTaq enzyme, 2 µl DNA template, and 17 µl ddH_2_O to make the final volume 25 µl. The PCR amplification program was designed as follows: initial denaturation at 94℃ for 5 min; 34 cycles of denaturation at 94℃ for 1 min, annealing at 55℃ for 1 min, extension at 72℃ for 2 min; and a final extension at 72℃ for 5 min. The PCR amplicons were visualized on a 1.5% agarose gel. To reduce the randomness of the PCR reaction, each template was subjected to five parallel PCR reactions, and all the products were mixed for further study. The PCR product portions of all samples were multiplexed into a single pool using equal molecular weights and were concomitantly purified using the solid phase reversible immobilization (SPRI) based method of the TaKaRa MiniBEST Agarose Gel DNA Extraction Kit Ver.4.0 (Takara Biotechnology Co., Ltd). Sequencing libraries were generated using TruSeq^®^ DNA PCR‐Free Sample Preparation Kit (Illumina, USA) following manufacturer's recommendations, and index codes were added. The library quality was assessed on the Qubit@ 2.0 Fluorometer (Thermo Scientific) and Agilent Bioanalyzer 2100 system. At last, the library was sequenced on an Illumina HiSeq 2500 platform and 250 bp‐end reads were generated.

Raw sequences were processed in QIIME1.7.0 (Caporaso, Bittinger, et al., 2010). Sequences were quality trimmed into operational taxonomic units (OTUs) at a 97% identity Threshold (Edgar., 2010). Representative sequences from individual OTUs were then aligned against the Green genes corset (DeSantis et al. 2006) using PyNAST (Caporaso, Kuczynski, et al., 2010). Taxonomic assignment was carried out with the RDP classifier (Wang et al., 2007). Resampling for each sample according to the minimum sequence numbers was performed before the downstream analyses.

### 2.5 Sequence data preparation and analyses

Paired‐end reads were assigned to samples based on their unique barcode and truncated by cutting off the barcode and primer sequence. Paired‐end reads were merged using FLASH (Magoc & Salzberg, 2011), a very fast and accurate analysis tool, designed to merge paired‐end reads when at least some of the reads overlap the read generated from the opposite end of the same DNA fragment, and the splicing sequences were called raw tags.

### 2.6 Biogenic amines determination

The analysis methods and HPLC systems for biogenic amines determination were described in a previous research paper (Qiao et al., 2020).

All biogenic amine standards, histamine dihydrochloride, tyramine dihydrochloride, putrescine dihydrochloride, and cadaverine dihydrochloride were purchased from Wako (Tokyo, Japan). The standard amines (50 mg) were dissolved in 0.1 M HCl. The standard amine solutions were diluted by 0.1 M HCl (each at 10 μg/ml) and used as the standard stock solution, respectively.

Each fish sample of 5 g was homogenized with 50 ml of 20% trichloroacetic acid in centrifuge tube for 10 min at room temperature. The homogenates were centrifuged (8000*g*, 10 min, 4°C) and filtered with 0.2 μm membrane filter paper (Wako, Japan), and 10 ml supernatant was diluted to 100 ml with distilled water. Standard amine samples 5 ml and fish samples 5 ml were derivatized with dansyl chloride. A derivatized standard solution and a derivatized fish sample solution 10 μl were used for HPLC analysis.

The content of amines in the samples were determined with a liquid chromatography (Shimadzu, Tokyo, Japan) consisting of a PU‐980 pump, low pressure gradient unit LG‐980–02, Fluorescence detector RF‐550, A Symmetry C18 Waters column (5 μm, 250 mm × 4.6 mm, Waters, USA). The gradient elution program begins with solvent A 6:4 (V/V) acetonitrile: water at a flow rate of 1.3 ml/min for the first 30 min, followed by a linear increase solvent A: solvent B (acetonitrile) 0:100 for 20 min, further solvent A: solvent B (acetonitrile) 0:100 held constant for 10 min with excitation wavelength 325 nm and fluorescence wavelength 525 nm.

Standard biogenic amines and fish samples were analyzed together. A standard solution was also analyzed intermittently between fish samples to check chromatographic consistency. Each sample was analyzed for triplicate. The peak heights of the standard biogenic amine solutions were used to prepare standard curves and then for determination of the amine content in the fish samples.

### 2.7 Statistical analysis

All experiments were performed in triplicate. All statistical analyses were performed using the SPSS statistical program (Version on 21, IBM Co.). And a value of *p* < .05 was considered as statistically significant. Data were expressed as mean ± standard deviation (*n* = 3).

## 3 RESULTS AND DISCUSSION

### 3.1 Results

#### 3.1.1 Microbiological analyses

##### Plate count agar media for microbiology analysis

**FIGURE 1:**
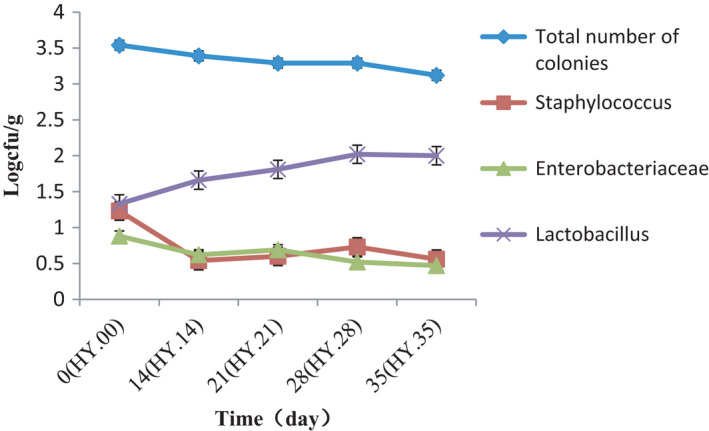
The number of total bacteria, *staphylococci, lactic acid bacteria, Escherichia coli* following with storage time

Bacterial counts were calculated on plate count agar media, and the results are shown in Figure 1. The total number of colonies decreased slowly following with the increase of storage time and ranged from 3.54 to 3.12 log cfu/g from 0 day to 35 days. *Staphylococcus* and *Enterobacteriaceae* counts were approximately the same, and *Staphylococcus* and *Enterobacteriaceae* appeared in a decreasing trend. *Staphylococcus* was 1.23log cfu/g at the 0 day and decreasing to 0.56log cfu/g at 35 days, and *Enterobacteriaceae* was 0.88log cfu/g at the 0 day and decreasing to 0.47logcfu/g at 35 days. While because the *Lactobacillus* are the dominant bacteria in pickled products, so they showed an increasing trend, but the increasing rate of *Lactobacillus* was relatively low.

#### 3.1.2 Analysis of bacterial diversity by high‐throughput DNA sequencing

##### Species taxonomy and abundance distribution analysis

The V3‐V4 region sequence reads of 16S rDNA were obtained by the sequencing of the amplicon pool. After filtering low‐quality reads, a total of 192,762 remained. To facilitate a quick and comprehensive understanding of the OTU clustering and annotation of each sample, the OTU clustering and annotation results of each sample were comprehensively analyzed (Table. 1). It indicated that the richness of the samples varied from a minimum of 55 (HY.14) to a maximum of 398 (HY.21) OTUs. As a result of this screening step, 222,762 high‐quality sequences were used for subsequent analysis.

**TABLE 1:** OUTs and estimation of species richness

Sample	Observed species	Sequence(*n*)	Reads OTU(*n*)	ACE	Chao estimator	Shannon index	Simpson index
HY.00	112	46,359	136	145.73	132.67	2.12	0.58
HY.14	53	47,621	55	56.17	54.11	1.57	0.39
HY.21	377	42,229	398	402.83	396.8	4.31	0.8
HY.28	276	53,221	306	333.91	328.98	3.57	0.77
HY.35	182	33,332	200	209.12	202.32	2.59	0.59

**FIGURE 2:**
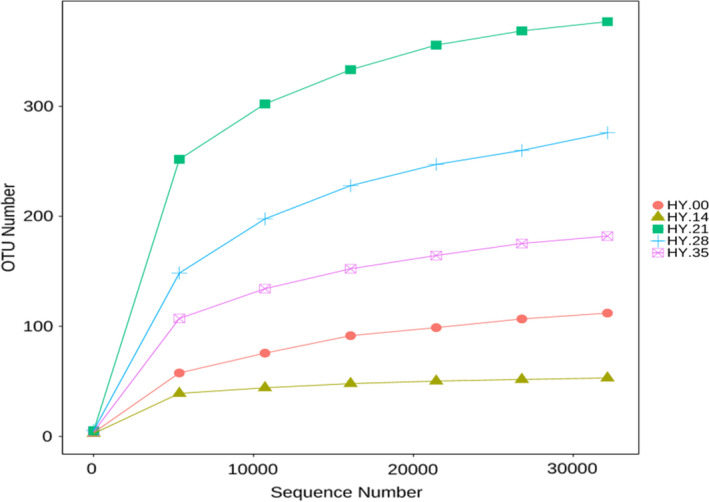
Rarefaction curves analysis of bacteria of the salted fish sample

The rarefaction curve, which is a form of sample dilution curve, was constructed by the extracted sequencing data and the corresponding number of species (OTUs number) (Figure 2). The five‐curve tended to be flat, reflecting the rationality of sequencing data and that more data will only produce a small number of new species (OTUs).

The α‐diversity of the bacteria in the salted fishes was analyzed (Table 1). The ACE estimator demonstrated the difference between 56.17 (HY.14) and 402.83 (HY.21) of the potential numbers of OTUs in the samples, and similar analysis data were obtained with the chao richness estimator (54.11–396.8), indicating a significant variation in bacterial richness. And HY.21 had the highest Shannon index (4.31), and the second Shannon index was HY.28 (3.57), and the lowest Shannon index was 1.57, indicating the highest bacteria diversity was HY.21 and the lowest bacteria diversity was HY.14.

##### Structure and abundance analysis of the flora

**FIGURE 3:**
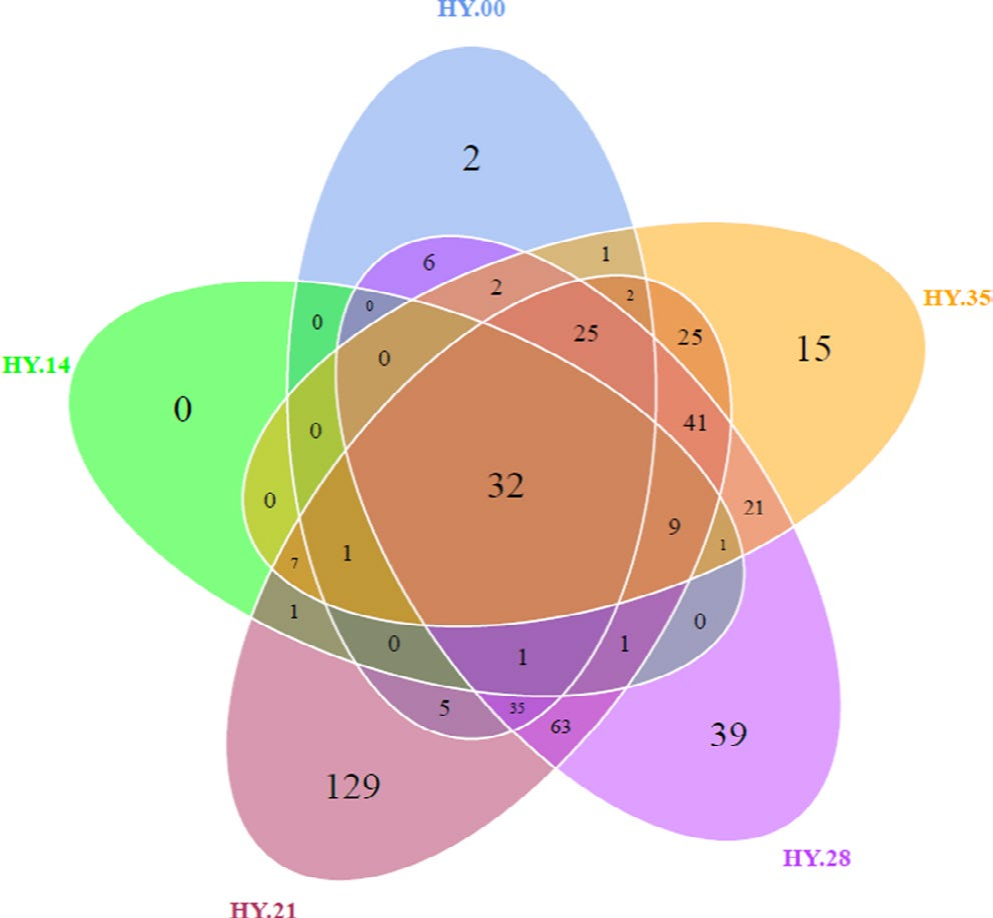
Venn analysis of microbial community of the salted fish samples following with storage time

Venn diagram can be used to count the number of OTUs that are common and unique to multiple samples. The OTU with a similarity of 97% was selected for this study. The results are shown in Figure 3, which can directly show the similarity and overlap of OTU compositions among apparent samples. 32 common OTUs in the five samples were shown in the Figure 3 of the Venn analysis, and the highest of 377 OTUs were found in the sample of HY.21, and the lowest OTUs of 53 were found in the sample of HY.14. The number of OTUs from the cluster showed some differences for samples at different times, and the number was basically HY28 >HY21 >HY35>HY00 >HY14 (*p* < .05).

**FIGURE 4:**
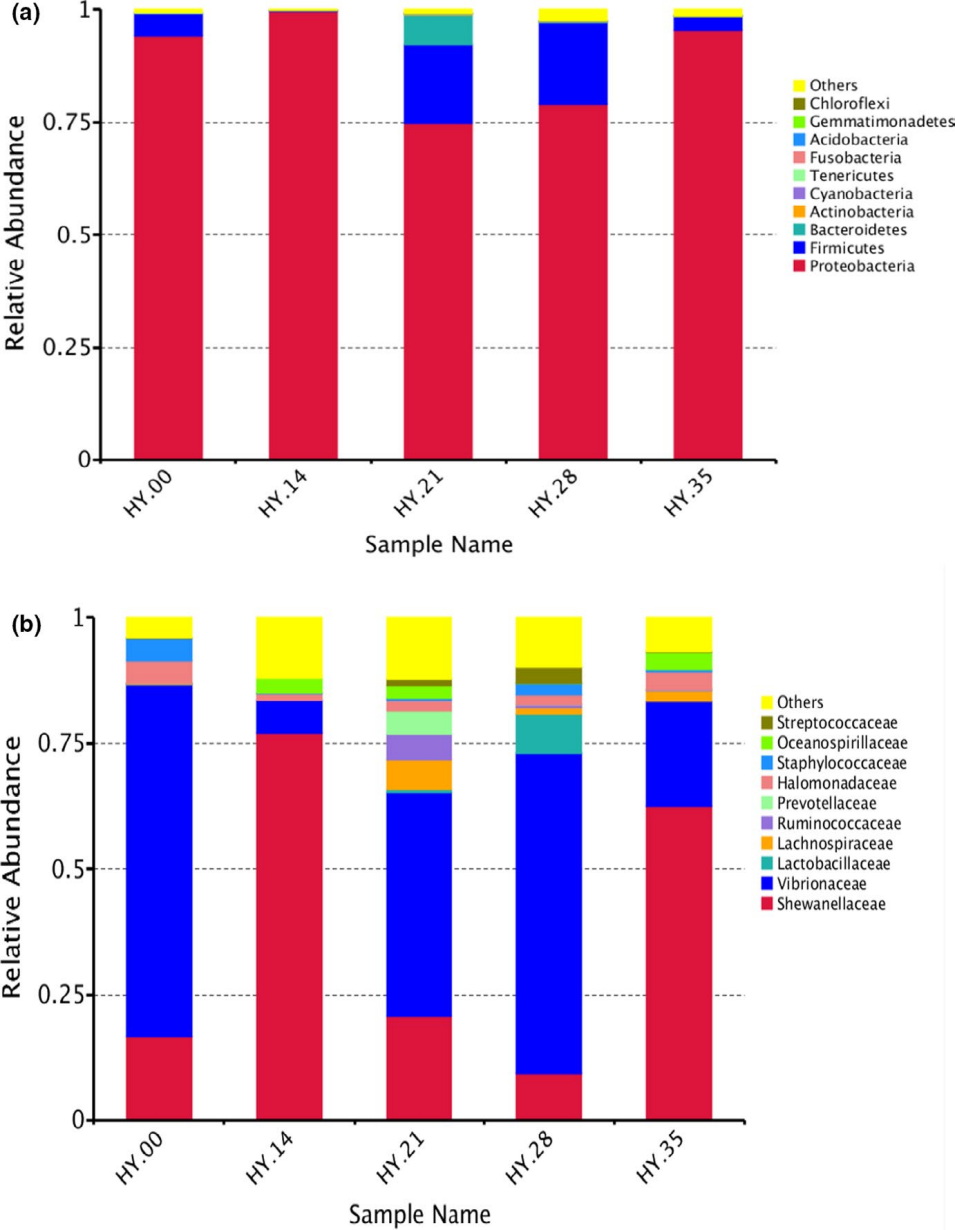
Relative abundance of bacteria of salted fish samples (a) phylum level (b) genus level

All samples of the sequences were clustered which were based on the identified OTU with 97% sequence similarity for in‐depth analysis. At the 16Sr RNA phylum level, 11 dominant microphytes were detected in the five samples (Figure 4a). *Proteobacteria* is the most dominant phylum, accounting for 94.9%, followed by *Firmicutes,* accounting for 4.76% of the total phyla. The most dominant phylum of *Proteobacteria* was presented in HY.21, and the most dominant phylum of *Firmicutes* was presented in HY.28. The other phylum such as *Actinobacteria* and *Bacterioidetes* was also commonly presented in these samples at relatively lower abundance. And Figure 4b of the genus level showed similar trends. *Shewanellaceae* and *Vibrionaceae* were dominant, and *Shewanellaceae* was accounting for 42.7%, *Vibrionaceae* was accounting for 46.1%. However, the dominant *Lactobacillus* which often appears in pickled products was the highest in salted fish sample of HY.28.

**FIGURE 5:**
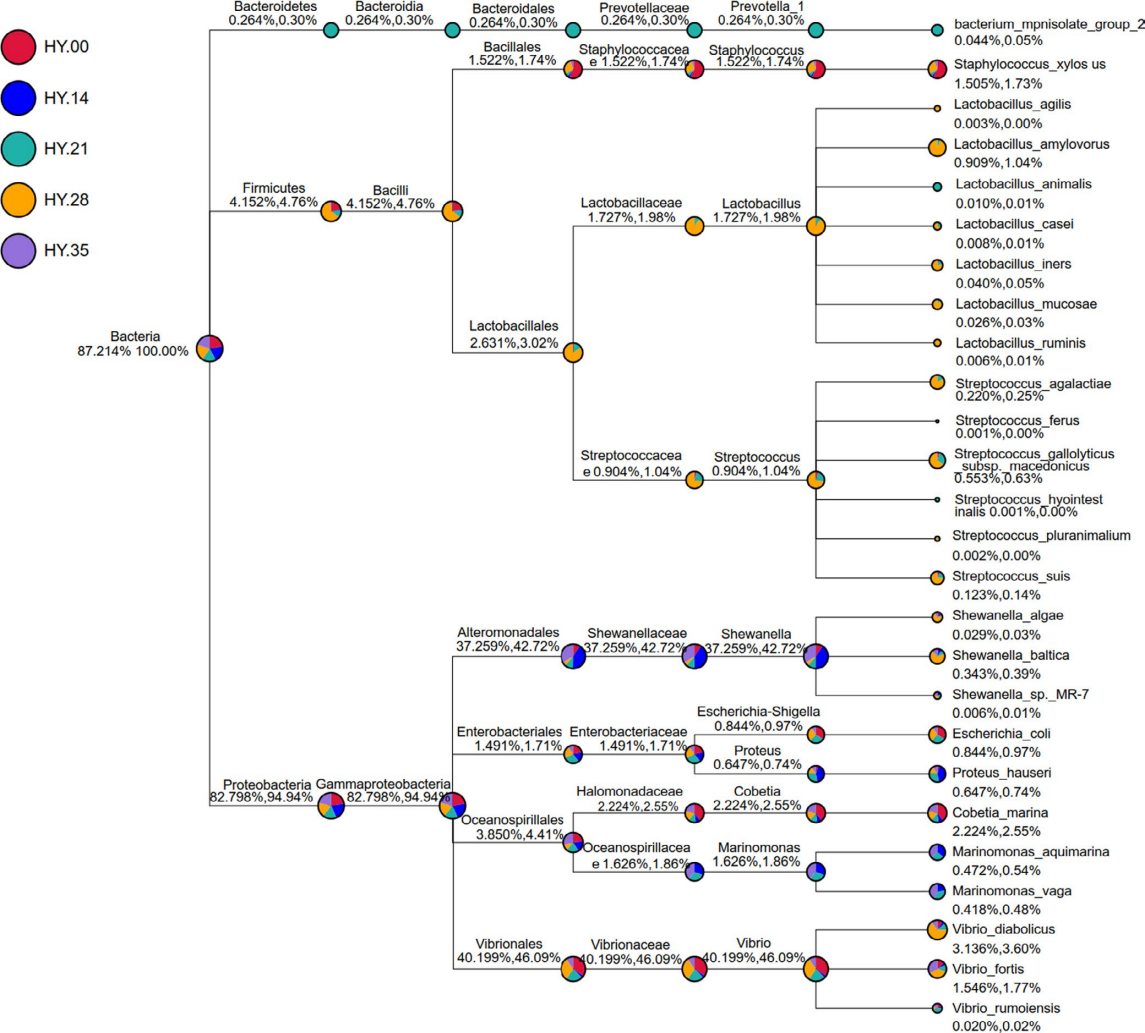
Tree species classification of the salted fish samples. Different colors of circles indicated different levels of classification, the size of the circle represented the relative abundance of the classification, two digital classification below were indicated relative abundance percentage, the percentage of all the former said the classification and classification of the species in the sample, while the latter represented the percentage of the species classification accounted for the selected samples

**FIGURE 6:**
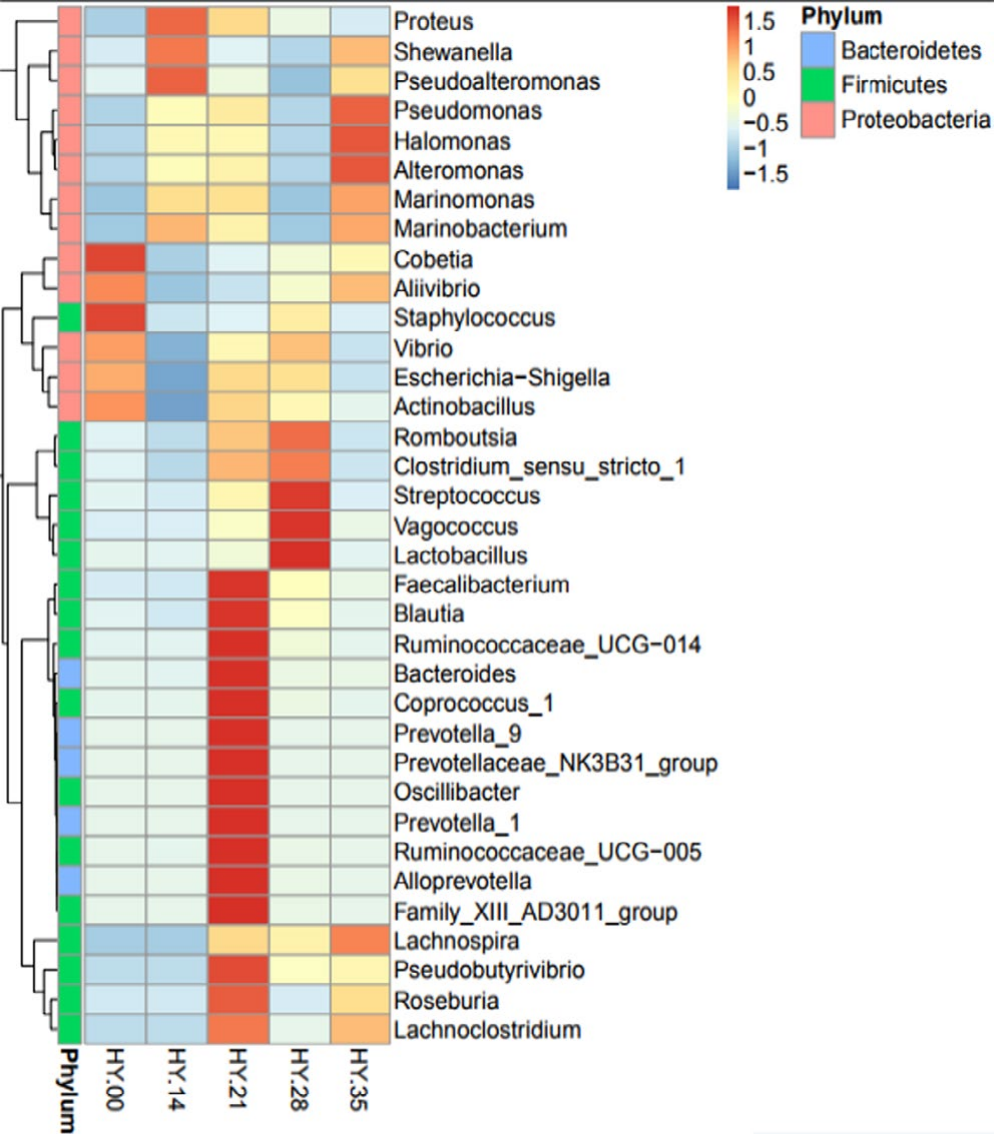
Heatmap of bacterial genera based on relative abundance of salted fish samples following with storage time

The detail of species classification and relative abundance percentage of the salted fish samples following with storage time were shown in Figure 5, and the salted fish samples were dominated by two main bacterial phylum ascribed to *Proteobacteria* (90.36% in HY.00, 98.80% in HY.14, 74.10% in HY.21, 78.31% in HY.28 and 88.55% in HY.35), and *Firmicutes* (4.82% in HY.00, 0.60% in HY.14, 17.47% in HY.21, 18.67% in HY.28, and 3.01% in HY.35), followed by *Bacteroidetes* (1.2% in HY.00, 1.0% in HY.14, 6.02% in HY.21, 1.5% in 28 HY.28 and 0.5% in HY.35, respectively), although with low relative abundance (Figure 6). The dominated *staphylococcus*, *lactobacillus,* and *streptococcus* belonged to *Firmicutes,* and *dominated Shewanellaceae, Enterobacteriaceae, Halomonadaceae,* and *Vibrionaceae* belonged to *Proteobacteria*.

##### The content of biogenic amines

**FIGURE 7:**
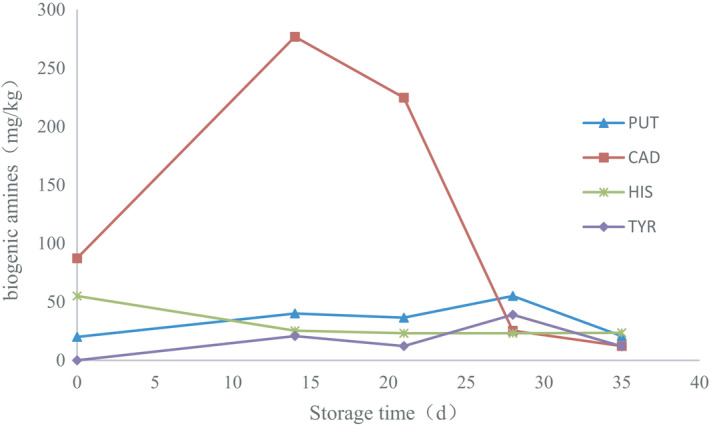
Variation trend of biogenic amines following with storage time

The contents of biogenic amines are shown in the Table 2 and Figure 7. The total amount of biogenic amines was 162.16 mg/kg in the sample of HY.00, and the total biogenic amines increased following with storing time gradually, up to maximum amount of 363.01 mg/kg in the sample of HY.14, and reached next maximum amount of 296.52 mg/kg in the sample of HY.21, and went down to 142.5 mg/kg in the sample of HY.28 and 68.42 mg/kg in the sample of HY.35, and cadaverine, putrescine, and tyramine first increased and then decreased. Among them, the variety of cadaverine was obvious, on the 0th, 14th, 21st and 28th, 35th days of storage; cadaverine was 87.36, 276.89, 224.76, 25.33, 12.17 mg/kg, respectively. Putrescine was 20.00, 40.05, 36.44, 55.04, 20.74 mg/kg on the 0th, 14th, 21st and 28th, 35th days of storage. The tyramine was smallest amount of production, and the largest amount was 38.99 mg/kg on 28th days, and the smallest amount was 11.97 mg/kg on 35th days. The variation trend of the content of putrescine and tyramine was basically the same. Nevertheless, the amounts of histamine decreased gradually following with storage time, the largest amount was 55.04 mg/kg on the 0th day and the smallest amount was 23.14 mg/kg on 28th days of storage, and there was little change in histamine on 21st and 28th, 35th days of storage.

**TABLE 2:** Content of biogenic amines in the samples

Sample	Biogenic amines			
	Putrescine (mg/kg)	Cadaverine (mg/kg)	Histamine (mg/kg)	Tyramine (mg/kg)
HY.00	20.00 ± 0.02	87.36 ± 0.17	55.04 ± 0.21	‐
HY.14	40.05 ± 0.11	276.89 ± 0.23	25.33 ± 0.14	20.74 ± 0.12
HY.21	36.44 ± 0.13	224.76 ± 0.19	23.15 ± 0.16	12.17 ± 0.05
HY.28	55.04 ± 0.03	25.33 ± 0.18	23.14 ± 0.24	38.99 ± 0.20
HY.35	20.74 ± 0.09	12.17 ± 0.07	23.54 ± 0.08	11.97 ± 0.08

Note

“‐”: Not detected.

Each result was measured three times to calculate the average value.

The values are expressed as averages of the average value ± *SD* (*p* < .05).

## 4 DISCUSSION

The analysis of bacterial communities involved in the salted fish was carried out by the microbiological and molecular method of HTS, and at the same time, variation of biogenic amines was also analysis using HPLC methods, and it is helpful for understanding the interaction between the microbial floras and biogenic amines. Change rules of bacterial counts on plate count agar media were consistent with the result of HTS, *Staphylococcus* and *Enterobacteriaceae* appeared in a decreasing trend, and the *Lactobacillus* of the dominant bacteria in pickled products showed an increasing trend.

Bacterial structure in the salted fish samples was dominated by two main bacterial phyla ascribed to *Proteobacteria* and *Firmicutes*. The clustering results showed that *Shewanellaceae* (37.26% of total sequences) and *Vibronaceae* of *Proteobacteria* (40.20% of total sequences) were the dominant microorganisms. *Firmicutes* were the second most dominant bacteria, and *Lactobacillus* belonging to *Firmicutes* mainly produce propionic acid, butyric acid, lactic acid, organic acid, and other amino acids that play an important role in product flavor and human health (Bianchi et al., 2011), while *Lactobacillus*, *Streptococcus,* and *Staphylococcus* of *Firmicutes* are the main bacteria in the fermented product and affected the flavor of salted fishes, and they also produce biogenic amines (Liu et al., 2016; Papageorgiou et al., 2018).

The influence of microbial community succession on the production and accumulation of biogenic amines in the fish samples following with storing time was studied. All the biogenic amines were initial increase and the decrease afterward following with storage time in this study, and the change of cadaverine was obvious, the content of cadaverine was 276.89 mg/kg of the highest level on the 14th day. And the accumulated amount of putrescine and tyramine were low level, the change trend was basically same. Among them, histamine showed a slow decrease trend, and 55.04 mg/kg of histamine in the sample of HY.00 exceeded 50 mg/kg of FDA guideline (FDA, 1996). And 23 mg/kg of histamine remained unchanged in the samples from 14 days to 35 days elementary, and the accumulation of histamine and other biogenic amine was consistent with the dominant flora of reported the biogenic amine producer and decomposer of *staphylococcus* and *Escherichia* in the samples of HY.00 after 12 hr pickling (Kim et al., 2009). The amount of biogenic amines was related to dominant bacteria of *Prevotellaceae* in the sample of HY.14, although no paper has reported the ability of *Prevotellaceae* to produce biogenic amines, and screened *Prevotellaceae* in this study has the ability to producing biogenic amines, mainly cadaverine and tyrosine (data not shown). And dominant bacteria of *Shewanella*, *Proteus,* in the sample of HY.21 can produce the biogenic amines (Ge et al., 2017; Helinck et al., 2013; Wang et al., 2019). And *Lactobacillus*, *Vibrionaceae*, and *Staphylococcus* dominated in the sample of HY.28, and there were also dominant amine‐producing strains in *Vibrionaceae* (Landete et al., 2007). And on the one hand, lactic acid bacteria have the ability to produce amine; on the other hand, lactic acid bacteria can degrades biogenic amines, mainly degrades putrescine and cadaverine. And *Staphylococcus* also degrades biogenic amines. Resulting in the gradual decrease of amount of biogenic amines (Zaman et al., 2010), because of the presence of dominant lactic acid bacteria from 28th day, lactic acid bacteria and its metabolites can inhibit the growth of other microorganisms, moreover, because of the influence of low temperature and low salt, so the microbial population and microbial metabolites decrease gradually, and the content of biogenic amines decrease gradually. The change of bacterial flora leads to the increase and then decrease of amount of biogenic amines following with the storage time, the complex bacteria were affected by low temperature, salt concentration, and metabolic products of the bacteria, the large numbers of experiments are needed to further study the interactions between bacterial flora and biogenic amines. And further analyze the influence of microbial structure on the quality of salted fish.

## 5 CONCLUSION

The change rule of microbial diversity and BAs content were studied during duration of storage period in salted fishes of one‐night courtyard in China. Two factors including salinity and structure of flora affect the safety and quality on salted fish product. Most of biogenic amines decreased after 14 days following with the changes in flora and salt penetration, but the biogenic amines in salted fish are not ignored. Microbes give special flavor for salted fish, but form the biogenic amines at the same time. The interaction was preliminary analysis between bacterial diversity and biogenic amines production in a salted mackerel in the study. So microbes should be controlled in the process of processing salted fishes. Future studies will focus on interactions between bacterial flora and biogenic amines by detail experiment.

## CONFLICT OF INTEREST

Zhihua Tao, Weiqi Liu, Qinxia Hu, Xue Wu, Shuying Xie, Hongmei Zhang, and Minghui Fu declare that they have no conflict of interest.

## AUTHOR CONTRIBUTIONS

**Zhihua Tao:** Formal analysis (equal); Funding acquisition (equal); Investigation (equal); Writing‐original draft (equal); Writing‐review & editing (equal). **Weiqi Liu:** Investigation (equal); Methodology (equal); Software (equal). **Qinxia Hu:** Data curation (equal); Methodology (equal). **Xue Wu:** Resources (equal); Visualization (equal). **Shuying Xie:** Supervision (equal). **Hongmei Zhang:** Validation (equal); Visualization (equal). **Minghui Fu:** Resources (equal); Software (equal). **Yang Jiang:** Investigation (equal); Writing‐original draft (equal).

## ETHICAL STATEMENT

This article does not contain any studies with human participants or animals performed by any of the authors.

## Data Availability

All data included in this study are available upon request by contact with the corresponding author.
